# Stable n‑Type Conduction in WO_*x*_‑CNT Hybrid Films

**DOI:** 10.1021/acsaelm.5c01933

**Published:** 2025-11-17

**Authors:** Ayesha Farooq, Luca Bignardi, Matus Stredansky, Marco Caputo, Sharath Sasikumar, Ferdinando Bassato, Regina Ciancio, Simone Dal Zilio, Andrea Goldoni, Paolo Piseri, Tommaso Mazza, Silvia Rubini, Cinzia Cepek

**Affiliations:** † CNR - Istituto Officina dei Materiali (IOM), 518735 AREA Science Park, Basovizza, 34149 Trieste, Italy; ‡ Department of Physics, 9315 University of Trieste, via Valerio 2, 34127 Trieste, Italy; § Elettra Sincrotrone Trieste, 518735 AREA Science Park, Basovizza, 34149 Trieste, Italy; ∥ European XFEL, Holzkoppel 4, 22869 Schenefeld, Germany; ⊥ 518735 AREA Science Park, Padriciano 99, 34149 Trieste, Italy; # Dipartimento di Fisica “Aldo Pontremoli”, Università degli Studi di Milano, via Celoria 16, 20133 Milano, Italy

**Keywords:** tungsten oxide nanoclusters, carbon nanotube hybrids, hybrid nanostructures, supersonic cluster beam deposition, gas sensing, n-type conduction

## Abstract

Nanostructured hybrid films composed of tungsten oxide (WO_*x*_) nanoclusters and vertically aligned carbon nanotubes (CNTs) were synthesized through a combination of chemical vapor deposition and supersonic cluster beam deposition. The use of a cluster source enabled the direct fabrication of oxygen-deficient, nonstoichiometric WO_*x*_ nanoclusters, which decorated the CNT sidewalls with a characteristic “beaded necklace-style” morphology. Electrical resistance measurements under ethanol exposure in ultrahigh vacuum revealed a distinct behavior consistent with n-type conduction, unlike the intrinsic p-type behavior of pristine CNTs and of WO_*x*_ films. This inversion is linked to the appearance of an interfacial charge transfer from the oxygen vacancies in the defective WO_*x*_ nanoclusters to the CNTs, which injects electrons into the CNT network and shifting its Fermi level, thereby inverting the conduction type. Notably, this n-type conduction response remained stable even after prolonged air exposure. These results propose a viable approach to achieving air-stable n-type doping in CNT-based nanostructures.

## Graphical abstract

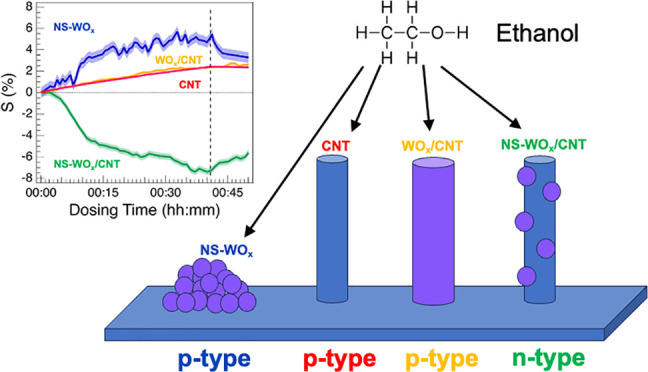

## Introduction

Hybrid materials represent a broad family of systems in which the integration of distinct components enables multifunctional properties that exceed those of the individual constituents.^1,2^ Within this context, carbon-based nanomaterials have emerged as a particularly versatile and widely explored platform, often serving as the structural and electronic backbone of advanced hybrid architectures. Among them, carbon nanotubes (CNTs) stand out for their exceptional mechanical strength, high electrical conductivity, and large specific surface area. They also provide robust and flexible scaffolds for hybridization with other nanomaterials, unlocking complementary functionalities such as catalytic activity, light responsiveness, and tunable charge transport.^3–6^ Moreover, their quasi-one-dimensional geometry supports ballistic electron transport over micrometer-scale distances, making CNTs highly attractive building blocks for next-generation electronic devices and sensors.^7–11^

Transition metal oxides have also become essential components in the development of functional hybrid systems, owing to their diverse chemical compositions, rich defect chemistry, and highly tunable electronic properties. Among these, tungsten oxides (WO_*x*_) stand out for the adaptable stoichiometry and electronic versatility, which can be finely tuned through oxygen vacancy engineering and controlled oxidation states. These characteristics make these oxides ideally suited for a broad range of applications, including gas sensing, photocatalysis, electrochromic devices, and energy storage technologies.^12–20^

Oxygen vacancies and interfacial donor states are recognized as key factors governing electronic behavior in transition-metal oxides. Greiner and co-workers^21^ demonstrated that defect-induced modifications in the oxide electronic structure facilitate charge-transfer processes, thereby enhancing n-type conductivity and catalytic activity. In a broader context, Tokura and Nagaosa^22^ highlighted that defects in transition-metal oxides can trigger orbital reconstruction and emergent charge-transport phenomena. Reports on WO_3_ align well with this framework, with several studies showing that oxygen vacancies drive electron-donor behavior, enhance carrier density, and promote interfacial charge transfer.^13,15,23^ These outcomes suggest that the defect concentrations can be a viable route to improving electronic transport in composite materials. When combined with CNTs, their intrinsic redox activity and oxygen-vacancy-driven sensing capabilities can be effectively coupled with the exceptional conductivity, high aspect ratio, and mechanical robustness of the carbon backbone. Such a combination enables hybrid systems with tailored interfacial chemistry, enhanced charge-transfer dynamics, and multifunctional behavior that would be difficult to achieve with either component alone.

Hybrid nanostructures that integrate CNTs with inorganic materials therefore offer unique opportunities to exploit synergistic effects and engineer advanced sensing platforms. Numerous studies have demonstrated that composites based on WO_*x*_ and CNTs exhibit superior gas-sensing performance compared to their individual constituents, showing higher sensitivity, improved selectivity, and faster response and recovery times.^15,24–34^ Although the precise origin of this enhanced performance is not yet fully understood, it is commonly attributed to the formation of p–n heterojunctions at the WO_*x*_-CNT interface, which facilitate efficient charge separation and modulate the electrical response of the sensor upon gas adsorption.^25,32,33^ Beyond electronic structure, the morphology and nanostructure of the oxide play a critical role: architectures such as nanorods, nanowires, and hierarchical assemblies dramatically increase the surface-to-volume ratio, provide abundant adsorption sites, and accelerate surface reactions.^14,16^

Interfacial effects are particularly significant in these hybrid systems, as interactions between WO_*x*_ and CNTs can lead to doping effects that fundamentally alter charge-carrier concentration and band alignment. Depending on the chemical environment and degree of oxide reduction, both p-type and n-type doping can occur, thereby allowing control over sensitivity, response direction, and recovery dynamics. This tunability enables efficient device operation even at room temperature, a major advantage over conventional metal oxide sensors that typically require elevated operating temperatures.^32^ Further studies have shown that WO_*x*_ clusters themselves can act as electron donors, transferring charge to the underlying CNTs. In particular, reduced WO_*x*_ species with a high density of oxygen vacancies are predicted to inject electrons into the CNT network, shifting its Fermi level and potentially inverting the conduction type relative to pristine CNTs.^33,35^ Despite these advances, maintaining the stability of such interfacial charge transfer under ambient conditions remains an significant challenge and a central focus of ongoing research.

Various methods have been employed to fabricate WO_*x*_-CNT hybrid nanostructures, enabling precise control over oxide morphology and distribution across the carbon nanotube network, which is crucial for tailoring their physical, chemical, and electronic properties. Beyond structural and functional optimization, several studies have emphasized that the interfacial characteristics between WO_*x*_ and CNTs are key to achieving reliable and sensitive gas sensing, particularly under ambient conditions.^24,26,31^

In this work, we investigated a novel WO_*x*_-CNT nanostructured hybrid prepared by depositing WO_*x*_ nanoclusters generated with a supersonic cluster beam deposition (SCBD) source onto vertically aligned CNTs grown by chemical vapor deposition (CVD) on a silicon wafer substrate. SCBD enabled the controlled decoration of the CNTs with WO_*x*_ nanoclusters, producing a distinctive “beaded necklace” morphology composed of discrete oxide nanoparticles anchored along the CNT walls—an architecture that, to the best of our knowledge, had not been previously reported. The synthesized hybrids were comprehensively characterized using in situ and ex situ techniques, including X-ray photoelectron spectroscopy (XPS), scanning electron microscopy (SEM), and transmission electron microscopy (TEM). Their chemical and electrical resistive responses to ethanol (EtOH) exposure in UHV were evaluated by monitoring resistance changes. We found that the response toward EtOH was radically different from that observed for pristine CNTs or for a WO_*x*_ layer grown on a Si wafer. Remarkably, the hybrids exhibited a behavior which is compatible with n-type conduction upon ethanol exposure, in stark contrast to the p-type response of pristine CNTs or a WO_*x*_ film on Si.

## Experimental Section

### Material Preparation and Characterization

Vertically aligned CNTs were synthesized via chemical vapor deposition (CVD) using acetylene as the carbon source and Fe (99.9% purity, 1.5 nm thickness) as the catalyst. The catalyst was deposited by electron-beam evaporation onto alumina films (thickness: 7.5 nm), which were themselves grown by DC magnetron sputtering (DCMS) on Si wafers. Prior to CNT growth, the substrate was degassed at 870 K and subsequently annealed in 100 sccm of H_2_ (grade 5) for 5 min. Growth was initiated by introducing a gas mixture of C_2_H_2_ (100 sccm, grade 2.5) and H_2_ (100 sccm, grade 5) at 870 K for 15 s. The growth pressure was maintained at approximately 10^–4^ mbar, with a base pressure of ∼10^–6^ mbar. The length of the resulting CNTs forest was approximately 5 μm.

Nanostructured WO_*x*_ (NS-WO_*x*_) clusters were synthesized at the INSPECT Laboratory (IOM-CNR, Trieste) using a supersonic cluster beam deposition (SCBD) source directly connected to the UHV chamber hosting both the photoemission station and the chamber for ethanol exposure.^36^ In the SCBD source, a tungsten rod (6mm diameter, 99.9% purity, EvoChem GmbH) was ablated by ion bombardment in an electric discharge ignited after the injection of an inert/O_2_ gas mixture (0–0.5% O_2_ in Ar, produced by dosing from two separate gas lines: high-purity Ar 6.0 and 20% O_2_ in He 6.0). The resulting plasma generated a supersonic beam of neutral WO_*x*_ clusters, which were focused by an aerodynamic lens and directed into the ultrahigh-vacuum chamber. The gas composition during deposition was monitored in real time using a residual gas analyzer (SRS RGA200) mounted on the UHV chamber.

DC magnetron sputtering (DCMS) deposition was performed using a tungsten target (99.95% purity) operated at 300 W DC power. Deposition was conducted in an Ar/O_2_ gas mixture (Ar: 20–23 sccm; O_2_: 3 sccm), yielding a deposition rate of approximately 0.4 nm s^–1^ at a base pressure of ∼10^–4^ mbar.

X-ray photoelectron spectroscopy (XPS) measurements were carried out at the INSPECT Laboratory using a Mg Kα source (*h*ν = 1253.6 eV) and a hemispherical PSP electron analyzer, with an overall energy resolution of approximately 0.8 eV. Spectra were collected in normal emission and referenced to the C 1s peak at 284.4 eV on the binding energy scale.^37^ Spectral fitting was performed using Doniach–Šunjic̀ line profiles combined with a Shirley-type background. The Lorentzian width was fixed to literature values,^37^ while Gaussian width, intensities, and peak positions were left as free parameters.

SEM images were acquired using ZEISS Supra 40 and LEO XB 1540 SEM-FIB scanning electron microscopes. TEM specimens were prepared by gently scratching the sample surface with a small blade and depositing the collected material onto carbon-coated copper grids. High-resolution TEM (HR/TEM) investigations were performed using a JEOL 2010 UHR field-emission gun microscope equipped with a field-emission Schottky cathode and operated at 200 kV with a spherical aberration coefficient *C*_s_ of 0.47 ± 0.01 mm, achieving a resolution of 0.19 nm under optimal phase-contrast imaging conditions.

### Ethanol Exposure and Resistive Response Measurements

A set of measurements of the resistive response upon ethanol exposure was carried out for each sample. These measurements were performed in ultrahigh vacuum (UHV, base pressure: 2 × 10^–10^ mbar) while keeping the sample at room temperature (300 K). Ethanol vapor was introduced into the UHV chamber at a pressure of 2 × 10^–5^ mbar (equivalent to approximately 20 ppb at 1 atm) via a leak valve connected to a dedicated stainless-steel reservoir. Prior to use, ethanol was degassed by several freeze–pump–thaw cycles. The vapor composition during exposure was monitored using a residual gas analyzer. The ethanol reservoir was maintained at approximately 370 K during dosing to achieve the desired vapor pressure in the chamber. Due to the low pumping speed of ethanol, pressure recovery after exposure was slow (approximately 90 min to return to 10^–7^ mbar), which limited postexposure recovery measurements.

In the resistive response experiments, two tantalum (Ta) electrodes (approximately 2 × 2 mm^2^ area) were gently brought into contact with the top surface of the investigated film, and the electrical resistance was measured in-plane through the overlayer film under UHV conditions. A schematic of the measuring geometry is reported in the Supporting Information, Figure S1. The contact resistance between the Ta pads and the film was minimized by fine-tuning the electrode pressure and monitoring the stability of the baseline resistance prior to gas exposure. Since the measurements report relative resistance changes rather than absolute conductivity values, any residual contact resistance does not affect the observed response trends. Electrical resistance was recorded using a precision multimeter connected to the tantalum contact pads. Baseline resistances ranged from ohms to megaohms, with less than 1% drift in the absence of ethanol.

The sensitivity (*S*) was defined as

$$S=\frac{\Delta R}{R_{0}}\times 100=\frac{R_{d}-R_{0}}{R_{0}}\times 100$$

where *R*_0_ is the initial resistance and *R*_d_ is the resistance measured during ethanol exposure. Assuming that Δ*R*/*R*_0_ was proportional to the number of adsorbed molecules, and given that pressure was proportional to dose, the resistance response as a function of dose could be approximated by an adsorption isotherm, as demonstrated in our previous work.^38^ Minor nonlinearities in *S* vs. time arise from small pressure fluctuations due to temperature variations in the EtOH reservoir.

## Results and Discussion

### Characterization of the Materials

As described in the Experimental Section, the NS-WO_*x*_/CNT hybrids were prepared by exposing the previously synthesized CNTs forest on Si wafers to a beam of WO_*x*_ clusters generated by the SCBD source. The morphology of the initial CNT carpet (434 ± 4 CNTs/μm^2^), as observed by SEM, is shown in Figure 1a.

**Figure 1:**
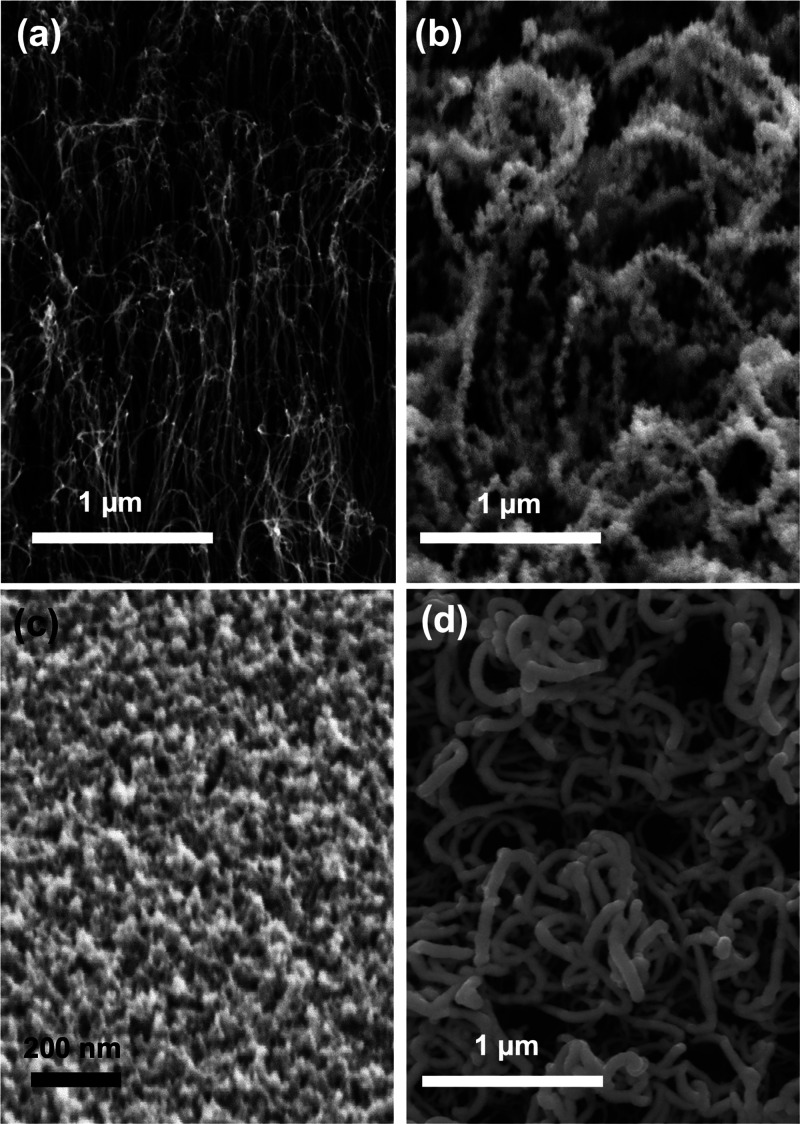
SEM images acquired on the various samples. (a) CNTs forest; (b) NS-WO_*x*_/CNT hybrid deposited by the supersonic cluster beam source; (c) 200 nm NS-WO_*x*_ layer on a Si wafer capped with native oxide; and (d) 25 nm WO_*x*_ layer deposited on a CNT forest by magnetron sputtering.

Figure 1b shows the SEM images acquired on the NS-WO_*x*_/CNT hybrids. The nanoclusters decorated the CNTs uniformly, adopting a “beaded necklace-style” nanostructured morphology without forming a continuous layer. This nanostructuring was also retained when clusters were deposited under the same conditions on a Si wafer (Figure 1c), producing a 200 nm-thick layer with a granular morphology. This reference sample was deposited simultaneously with the NS-WO_*x*_/CNT hybrid, ensuring the homogeneity of cluster production on both substrates.^39^ The last sample investigated was fabricated by depositing a 25 nm WO_*x*_ layer via DCMS on a CNT carpet similar to that used for the NS-WO_*x*_/CNT hybrids. Its morphology, as shown in Figure 1d, revealed that the oxide layer coated the CNTs uniformly while preserving their morphology at the micrometer scale.

It is important to remark that, to ensure homogeneous cluster coverage along the vertical height of the CNT carpet, we have selected CNT forests with a well-defined length and relatively low surface density, as specified earlier. This parameter proved crucial: excessively dense forests hinder the penetration of the cluster beam, leading to shadowing effects and incomplete decoration of the inner CNT walls. The chosen density guaranteed that the supersonic cluster beam could access the entire CNT length. As a control experiment, we deposited a 500 nm WO_*x*_ film by DCMS onto a much denser CNT forest (7.5 × 10^3^ CNTs/μm^2^). In this case (Figure S2, Supporting Information), a continuous microstructured oxide layer formed, while the vertical alignment of the CNTs was retained. This comparison highlights the crucial role of CNT density in determining the coating morphology. Based on these observations, we selected the CNT forest density that ensured a uniform nanocluster decoration along the entire nanotube length.

TEM investigations of the NS-WO_*x*_ material were carried out to determine the crystalline structure of the clusters obtained via SCBD, and the results are shown in Figure 2.

**Figure 2:**
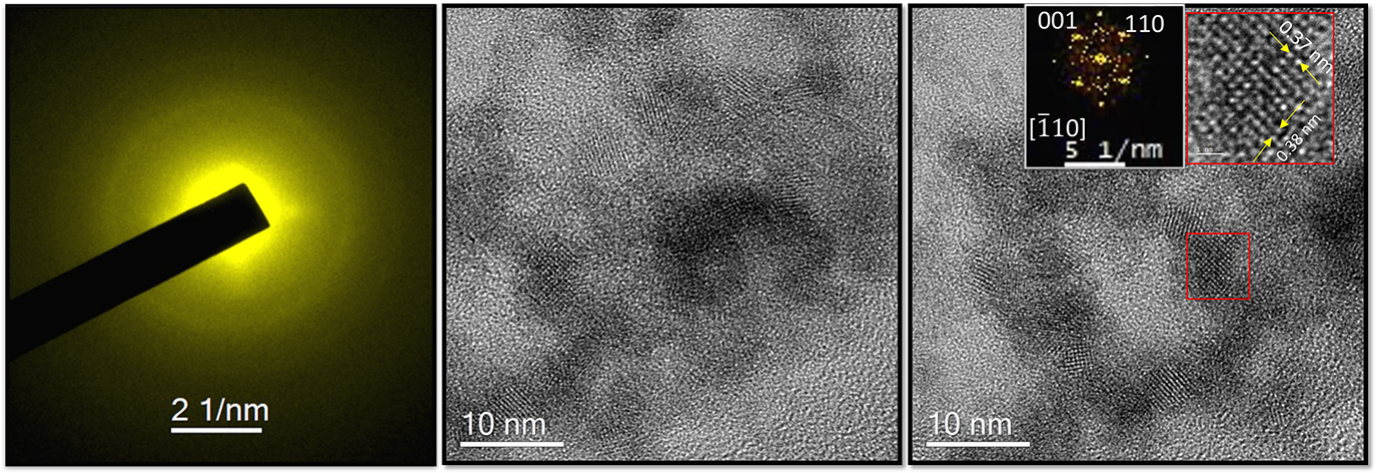
TEM characterization of the nanoparticle aggregates forming the NS-WO_*x*_ layer on a Si wafer. The left panel shows the selected-area electron diffraction (SAED) pattern acquired from the sample, indicating partial crystallinity. The central and right panels show TEM micrographs from different regions of the sample. The inset in the right panel presents a magnified view of the area highlighted in red, along with the corresponding fast Fourier transform (FFT) pattern.

The TEM images revealed the presence of nanoclusters exhibiting both amorphous and crystalline phases. Selected-area electron diffraction (SAED) patterns (left-hand panel of Figure 2) indicated rotational disorder but remained consistent with crystalline nanoparticles showing lattice spacings of 0.38 and 0.37 nm. These values correspond to the (001) and (110) planes of monoclinic WO_3_, as highlighted in the right panel of Figure 2.

Further insights into the chemical environment and interfacial interactions within the hybrid systems were obtained through X-ray photoelectron spectroscopy (XPS). A comparative analysis of the W 4f and O 1s core levels was performed on four representative samples: NS-WO_*x*_-CNT, NS-WO_*x*_ on a Si wafer with native oxide, a 25 nm WO_*x*_ film on CNTs, and a 200 nm WO_*x*_ film on a Si wafer with native oxide.

Figure 3a shows the W 4f core-level spectra. In all cases, the W 4f spectra displayed the characteristic spin–orbit doublet, with the W 4f_7/2_ component centered at a binding energy (BE) of 35.5 eV, consistent with tungsten in the W^6+^ oxidation state.^23,40^ However, a distinct shoulder at lower binding energy (BE = 35.0 eV) appeared exclusively in the NS-WO_*x*_-CNT and WO_*x*_-CNT samples prepared by DCMS. In contrast, this feature was absent in the planar 200 nm WO_*x*_/Si wafer sample, where the WO_*x*_-substrate interface lays beyond the probing depth of the measurement. We interpreted this shoulder as an electronic signature of interfacial state formation, a feature already observed for WO_3_-CNT systems.^26^ Its selective appearance in the CNT-containing architectures suggested a chemically and electronically active interface that was absent in the systems containing only WO_*x*_. Although the spectra were broad, the data obtained for the NS-WO_*x*_-CNT hybrid and the WO_*x*_-CNT samples could not be satisfactorily fitted using a single component with the same line shape adopted for the WO_*x*_ film on the Si wafer.

**Figure 3:**
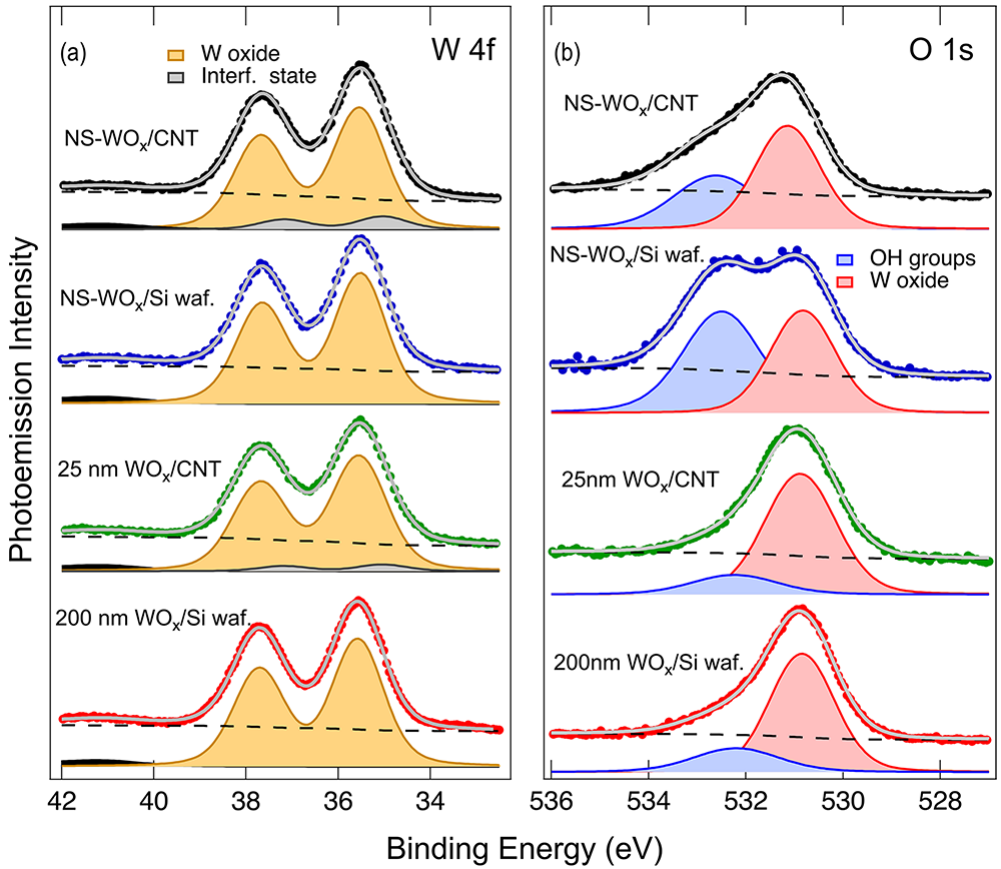
(a) W 4f and (b) O 1s spectra acquired on the different samples, as indicated in each panel. The filled colored dots represent the experimental data; the continuous gray line shows the best fit to the data using the spectral components (filled line profiles) and the background (dashed line) reported in each spectrum.

More details about the chemical composition of the samples were obtained by analyzing the O 1s core level, shown in Figure 3b. The O 1s spectra displayed two main components: one at BE = 530.8 eV, attributed to oxygen in WO_*x*_, and another at 532.5 eV, associated with surface hydroxyl groups formed upon air exposure. In the Si-wafer-supported samples, signals from the native SiO_2_ layer were not detectable due to the limited probing depth of the technique. Overall, the XPS data confirmed the comparable average stoichiometry of the WO_*x*_ phases obtained by different methods, while simultaneously revealing the formation of interface-specific electronic states exclusively in the CNT-based samples.

### Resistive Response to Ethanol

The resistive response to ethanol for the various samples is reported in Figure 4. Specifically, we plot the sensitivity (*S*), as defined in the Experimental Section, as a function of ethanol exposure time. In the following, we adopt the terminology of p-type and n-type conductivity to describe the observed changes in sensitivity upon ethanol exposure. These terms refer to the dominant charge carriers in the material, i.e., holes in p-type and electrons in n-type systems, and to how their concentration is modulated by interaction with gas molecules. Ethanol, being a reducing gas, typically donates electrons to the system. In a simplified picture, in p-type materials this electron donation decreases the hole concentration, leading to an increase in electrical resistance; conversely, in n-type materials, ethanol increases the electron concentration, resulting in a decrease in resistance. This framework is widely used to interpret the gas-sensing behavior of metal oxides, carbon nanostructures, and their hybrid systems when exposed to reducing or oxidizing species.^41,42^

**Figure 4:**
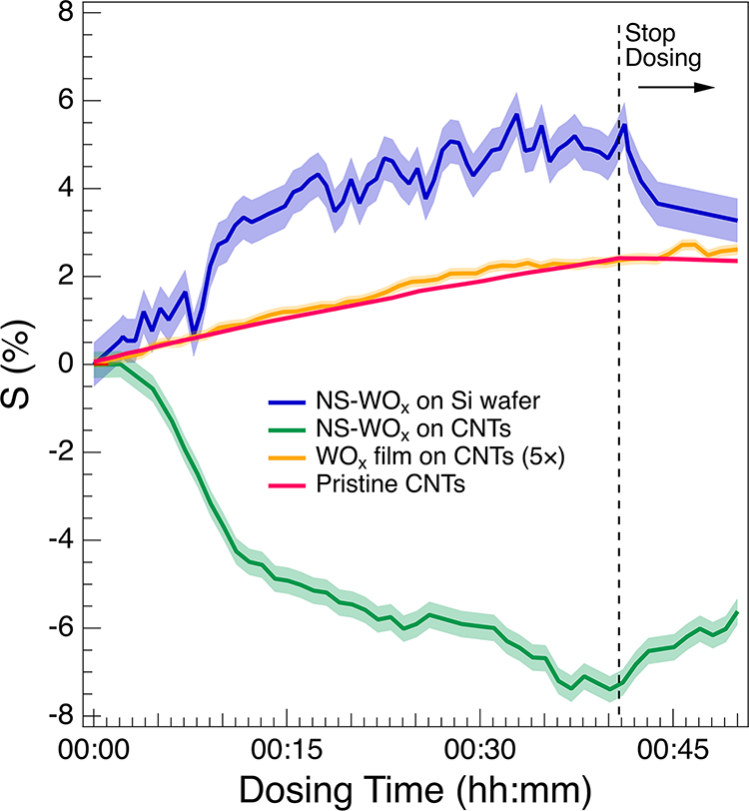
Sensitivity measurements of the various materials upon exposure to EtOH in UHV (partial pressure *p* = 2 × 10^–5^ mbar). The dashed line on the right indicates when EtOH exposure was stopped. The data for the WO_*x*_ film grown by DCMS on CNTs (yellow) were multiplied by 5 to show that they follow the same trend as the pristine CNT sample but with reduced intensity. Shaded regions represent the corresponding error margins.

We first discuss the behavior of the individual components, followed by that of the hybrids. For the pristine CNT forest, the resistance increased gradually during EtOH exposure—a feature typical of p-type semiconductors interacting with reducing gases^9^—and recovered once the flux was stopped. The maximum sensitivity observed in this work (*S* = 2.42 ± 0.05%), measured in UHV, was significantly higher than values reported under atmospheric conditions, where humidity reduces CNT sensitivity.^43^ The reproducibility of the measurement was verified (Figure S3, Supporting Information): repeating the experiment after a 24-h recovery period yielded identical response behavior. Furthermore, longer exposures (i.e., higher EtOH dose) led to higher final *S* values (5.5 ± 0.05%), indicating that the response remained well below saturation.

In contrast, the WO_*x*_ film grown on a Si wafer via DCMS showed only a modest and delayed resistance decrease of less than 1% (not shown), likely due to contact instabilities or limited adsorption sites. The WO_*x*_ film grown via DCMS on CNTs under the same conditions exhibited a much lower sensitivity (0.47 ± 0.03%), following a response curve similar to that of the CNTs but with attenuated amplitude (approximately five times smaller). This behavior likely resulted from passivation of the CNT surface by the continuous oxide coating, which suppressed the p-type conduction typical of CNTs.

The nanostructured WO_*x*_ (NS-WO_*x*_) films, however, exhibited markedly different behavior. A significantly enhanced response was observed for the NS-WO_*x*_ film grown via SCBD on Si wafers: at a total dose of 4.8 × 10^4^ L EtOH (after 40 min), the sensitivity reached approximately 5.4 ± 0.5%, outperforming the CNTs. The response rapidly recovered once EtOH exposure ceased. Although stoichiometric WO_3_ behaves as an n-type semiconductor at high temperatures,^26,44^ substoichiometric tungsten oxides can exhibit p-type conduction at room temperature.^45^ This behavior matched that of our NS-WO_*x*_/Si wafer sample and can be attributed to structural defects, visible in SEM and TEM, that accept electrons and enable hole conduction. It has been reported that sufficiently reduced WO_3–*x*_ achieves effective p-type behavior, supporting charge transport mechanisms distinct from those of stoichiometric WO_3_.^44^ Vacuum annealing these samples at 550 K for 10 min did not alter the XPS line shapes but reduced the sensitivity *S* from 5.5 to 1.4% (Figure S4), suggesting that oxygen defects govern the interaction with the reducing gas and that structural reordering upon annealing hinders this process.

The most striking behavior was observed for the NS-WO_*x*_-CNT hybrids. These displayed a negative resistance response, reaching a sensitivity of −7.3 ± 0.3% at the same EtOH dose as the other materials, with rapid onset and recovery. Such an inverted trend, consistent with n-type conduction, is rarely observed in CNT-based systems. We propose that this atypical behavior arises from interfacial electron transfer between the highly defective tungsten oxide nanoclusters and the underlying CNTs. This interpretation is supported by the contrasting behavior of the DCMS-grown WO_*x*_-CNT film, where a compact oxide layer suppresses such interactions, effectively passivating the CNT surface and hindering charge exchange.

A second series of sensitivity tests assessed pressure-dependent responses, relevant for sensing applications (Figure S5). These revealed an even higher sensitivity (*S* = – 14.0 ± 0.3%) when the NS-WO_*x*_-CNT hybrids were exposed to higher ethanol pressure (3 × 10^–4^ mbar). It is worth nothing that the initial phase of this second exposure was performed at the same pressure as the first set (Figure 4) and yielded a comparable response, confirming the persistence of the electronic structure modification at the hybrid interface. Remarkably, the sensitivity of our hybrids remained stable even after prolonged air exposure. The samples were synthesized, exposed to air for SEM and TEM characterization, and later reintroduced into UHV for resistivity measurements, maintaining performance after several weeks under ambient conditions. These characteristics underscore the potential of NS-WO_*x*_-CNT hybrids as reliable, air-stable n-type materials for CNT-based sensing and electronic devices.

A stable n-type conduction response in CNT-based hybrids at room temperature is both technologically valuable and fundamentally rare. While the measured resistance change represents the collective response of the hybrid film, the inverted sign indicates dominant n-type behavior induced by interfacial charge transfer. Most n-doping routes for CNTs lack stability under ambient conditions.^32^ Previous reports have shown that hybridization with transition-metal oxides can modulate the electronic properties of CNTs, notably by shifting their Fermi level and inducing n-type behavior, both for Mo^46^ and W oxides.^26,47^ As previously noted, oxygen-deficient WO_3‑x_ structures act as electron donors that transfer charge to adjacent CNTs through oxygen vacancies,^17,23,44,45^ effectively raising the CNT Fermi level and modifying their electronic structure. Experimental reports corroborate this mechanism,^15,23,41,45,47^ showing that oxygen vacancies in WO_3_ enhance both electrical conductivity and carrier density, thereby strengthening interfacial charge transfer. Our findings are consistent with this picture, indicating that interfacial charge transfer from oxygen-deficient nanostructured WO_*x*_ to CNTs induces robust n-type behavior.

Overall, WO_*x*_ nanoclusters introduce donor-like electronic states at the interface, creating favorable conditions for stable n-type conduction in our hybrids under the explored environmental conditions. Conversely, this behavior is absent in continuous oxide films, as observed for our DCMS WO_3_/CNT sample, underscoring the importance of a defective, nanostructured oxide phase in activating these interfacial electronic features.

## Conclusions

In this work, we demonstrated a link between morphology, interfacial chemistry, and charge transport in WO_*x*_/CNT hybrid nanostructures. By means of supersonic cluster beam deposition, we obtained a distinctive “beaded-necklace” arrangement of oxygen-deficient WO_*x*_ nanoclusters on vertically aligned CNTs, which induced upon ethanol response a reproducible inversion from the typical p-type response of CNTs to a stable behavior compatible with n-type conduction. The observed negative resistance variation, together with the interface-specific states revealed by XPS, highlighted the crucial role of oxygen vacancies and interfacial charge transfer in governing the electronic structure of the hybrids. Unlike conventional n-doping strategies for CNTs, which often lack ambient stability, the present approach achieved persistent n-type conduction even after prolonged air exposure. Our results, interpreted within the broader context of oxide/CNT hybrid literature, point to a general mechanism whereby oxygen-vacancy-rich oxide nanostructures establish donor-like interfacial states that inject electrons into the carbon network, shifting its Fermi level and inverting the conduction type. These findings contribute to the understanding of oxide–CNT interactions and provide a practical route for designing CNT-based platforms with controlled conduction type, offering a promising pathway toward robust, room-temperature sensors and emerging nanoelectronic architectures.

## Supplementary Material



## Data Availability

The authors have provided the data in the main manuscript and in the Supporting Information file. Any further data is available from the corresponding author upon reasonable request.

## Notes

Geometry of the contacts during resistive response measurements (Figure S1); SEM image of the FIB cross section of the 500 nm WO*_x_* layer deposited by DCMS on the CNT forest (Figure S2); exposure of a CNT-forest sample to EtOH in UHV (*p* = 2 × 10^–5^ mbar), to verify the reproducibility of the response and find the saturation dose (Figure S3); sensitivity response of the NS-WO_*x*_ deposited on the Si wafer during exposure to EtOH in UHV before and after thermal annealing (Figure S4); and exposure to EtOH of the NS-WO*_x_*/CNT hybrid in two different pressure regimes (Figure S5) (PDF)

## ∇

Department of Physics, COMSATS University Islamabad, Park Road, Tarlai Kalan, Islamabad 45550, Pakistan

## ○

School of Chemistry, The University of Birmingham, Edgbaston, Birmingham B15 2TT, U.K.

## ◆

Dipartimento di Fisica “Aldo Pontremoli”, Università degli Studi di Milano, via Celoria 16, 20133 Milano, Italy.
