# Supplementary material for: Stable n‑Type Conduction in WO x ‑CNT Hybrid Films
Source: ACS Appl Electron Mater. 2025 Nov 17;7(22):10438–45. doi: 10.1021/acsaelm.5c01933 (PMC12659432; doi:10.1021/acsaelm.5c01933)
Supplement: Supplementary file 1 [file el5c01933_si_001.pdf]

Supporting Information

Stable n-Type Conduction in WO_x-CNT Hybrid Films

Ayesha Farooq[a,b,1,§], Luca Bignardi[b,a,*,§], Matus Stredansky[a,2], Marco Caputo[c], Sharath Sasikumar[d,3], Ferdinando Bassato[a], Regina Ciancio[e], Simone Dal Zilio[a], Andrea Goldoni[c], Paolo Piseri[f], Tommaso Mazza[d], Silvia Rubini[a], and Cinzia Cepek[a*]

[a] CNR – Istituto Officina dei Materiali (IOM), AREA Science Park, 34149 Basovizza, Trieste, Italy
[b] Department of Physics, University of Trieste, via Valerio 2, 34127 Trieste, Italy.
[c] Elettra Sincrotrone Trieste, AREA Science Park, 34149 Basovizza, Trieste, Italy.
[d] European XFEL, Holzkoppel 4, 22869 Schenefeld, Germany.
[e] AREA Science Park, Padriciano 99, 34149 Basovizza, Trieste, Italy.
[f] Dipartimento di Fisica "Aldo Pontremoli" Università degli Studi di Milano, via Celoria 16, 20133, Milano, Italy.

[§] A.F and L.B. contributed equally to this work.
[1] Present Address: Dept. of Physics, COMSATS University Islamabad, Park Road, Tarlai Kalan, Islamabad 45550, Pakistan.
[2] Present Address: School of Chemistry, The University of Birmingham, Edgbaston, Birmingham, B15 2TT United Kingdom.
[3] Present Address: Dipartimento di Fisica "Aldo Pontremoli" Università degli Studi di Milano, via Celoria 16, 20133 Milano, Italy.

* Email: lbignardi@units.it (L.B.); cepek@iom.cnr.it (C.C)

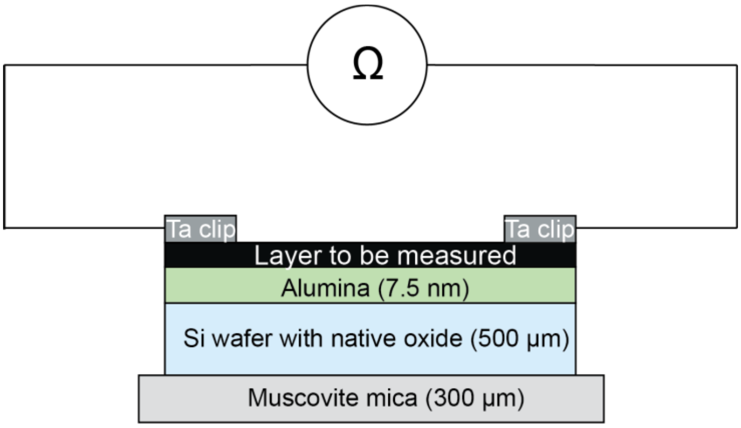

Figure S1. Geometry of the resistivity measurements performed under UHV conditions. Layer thicknesses are not drawn to scale. The thickness of the Ta clips was approximately 200 µm.

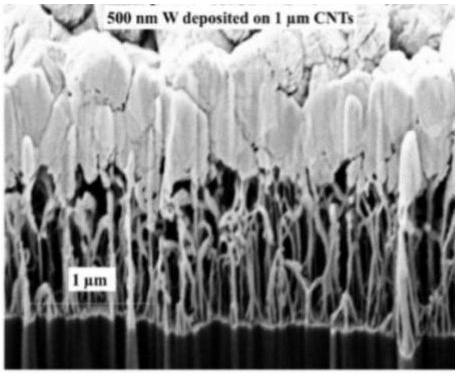

Figure S2. SEM image of FIB-cross section of 500 nm WO_x layer deposited by DCMS on CNTs forest. (CNTs density: $7.5x10^3$ CNTs/mm^2).

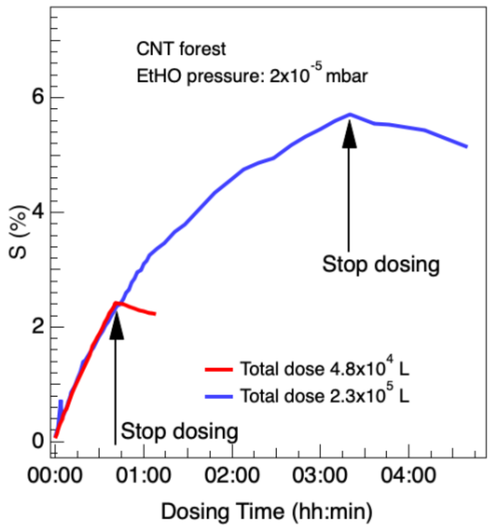

Figure S3. Exposure of a CNTs sample to EtOH in UHV (p=2x10^{-5} mbar), to verify the reproducibility of the data and find the saturation dose. The total amount of EtOH dosed is reported for each of the two exposures.

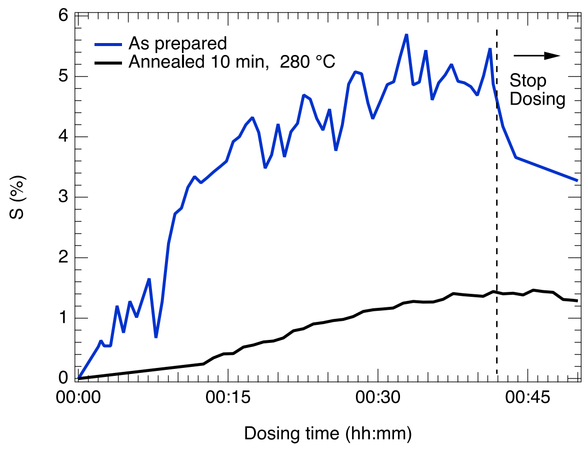

Figure S4. Sensitivity measurements for the NS-WO_x deposited on Si wafer during exposure to EtOH in UHV (partial pressure p=2×10^{-5} mbar) at RT (blue) and after annealing at 280 ˚C for 10 minutes in UHV (black).

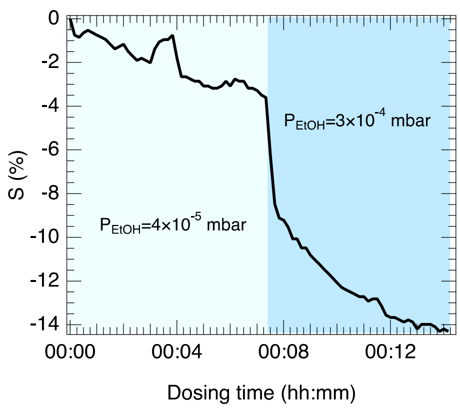

Figure S5. Exposure to EtOH of the NS-WO_x/CNTs hybrid in two different pressure regimes.
